# Genetic mapping and physiological analysis of chlorophyll-deficient mutant *in Brassica napus* L

**DOI:** 10.1186/s12870-022-03630-9

**Published:** 2022-05-18

**Authors:** Na Lin, Yumin Gao, Qingyuan Zhou, Xiaoke Ping, Jiana Li, Liezhao Liu, Jiaming Yin

**Affiliations:** 1 College of Agronomy and Biotechnology, Southwest University, Beibei, Chongqing, 400715 PR China; 2 Academy of Agricultural Sciences, Southwest University, Tiansheng Rd2#, Beibei, Chongqing, 400715 PR China

**Keywords:** *Brassica napus*, Leaf color, Mutant, Genetic mapping, Transcriptome

## Abstract

**Background:**

Leaf color mutants have reduced photosynthetic efficiency, which has severely negative impacts on crop growth and economic product yield. There are different chlorophyll mutants in *Arabidopsis* and crops that can be used for genetic control and molecular mechanism studies of chlorophyll biosynthesis, chloroplast development and photoefficiency. Chlorophyll mutants in *Brassica napus* are mostly used for mapping and location research but are rarely used for physiological research. The chlorophyll-deficient mutant in this experiment were both genetically mapped and physiologically analyzed.

**Results:**

In this study, yellow leaf mutant of *Brassica napus* L. mutated by ethyl methyl sulfone (EMS) had significantly lower chlorophyll a, b and carotenoid contents than the wild type, and the net photosynthetic efficiency, stomatal conductance and transpiration rate were all significantly reduced. The mutant had sparse chloroplast distribution and weak autofluorescence. The granule stacks were reduced, and the shape was extremely irregular, with more broken stromal lamella. Transcriptome data analysis enriched the differentially expressed genes mainly in phenylpropane and sugar metabolism. The mutant was mapped to a 2.72 Mb region on A01 by using BSA-Seq, and the region was validated by SSR markers.

**Conclusions:**

The mutant chlorophyll content and photosynthetic efficiency were significantly reduced compared with those of the wild type. Abnormal chloroplasts and thylakoids less connected to the stroma lamella appeared in the mutant. This work on the mutant will facilitate the process of cloning the *BnaA01.cd* gene and provide more genetic and physiological information concerning chloroplast development in *Brassica napus*.

**Supplementary Information:**

The online version contains supplementary material available at 10.1186/s12870-022-03630-9.

## Background

Photosynthesis is crucial in crop production and provides energy and carbohydrates for plant vegetative and productive growth. Pigments capture light energy and convert it into the chemical energy ATP and NADPH, which are used for CO_2_ fixation to synthesize carbohydrates. The primary photosynthetic pigment chlorophyll (Chl.) in plants is responsible for light harvesting and drives electron transport in reaction centers [1]. Chl. biosynthesis begins with glutamate to Chl. a and Chl. b and includes 20 different enzyme reactions [2]. Most Chl. biosynthetic genes have been cloned and validated in plants [2, 3]. Furthermore, the expression of key plant Chl. biosynthetic genes is tightly and coordinately controlled by transcription factors, which respond to environmental factors, including light signals, hormonal levels and nutritional supplies [4]. Mutation of biosynthetic genes or associated transcription factors introduces Chl. content variation and malformed chloroplasts and impairs photosynthetic efficiency.

There are different chlorophyll mutants in *Arabidopsis* and crops that can be used for genetic control and molecular mechanism studies of Chl. biosynthesis, chloroplast development and photoefficiency. In *Arabidopsis*, more than 27 genes responsible for Chl. b synthesis starting from glutamyl-tRNA have been identified[2, 5]. More than 70 chlorophyll mutants exhibiting albino, chlorina, stripe, virescent, yellow–green and zebra leaves were identified in rice [6]. Jung et al. established T-DNA pools for rice mutants, and 189 lines showed a chlorophyll-deficient phenotype that segregated as a single recessive locus in the T_2_ generation[7]. Zhao et al. identified that HD domain-containing proteins affect chlorophyll biosynthesis and chloroplast development in the white stripe leaf3 mutant [8]. Zhang et al. identified an incompletely dominant gene located on chromosome 2BS flanked by the simple sequence repeat marker Xwmc25 responsible for yellow leaf color [9]. Transcriptome analysis of yellow leaf color in wheat indicated that DEGs were involved in Chl. biosynthesis, carotenoid biosynthesis, photosynthesis, and carbon fixation [10]. Chlorophyll deficiency was also detected in barley and wheat [11–13], maize [14–17], soybean [18–22] and cucumber [23, 24].

Oilseed rape, *Brassica napus* L., an amphidiploid species formed by the natural hybridization of two diploid progenitors, *Brassica rapa* (AA, 2n = 20) and *Brassica oleracea* (CC, 2n = 18), provides 13%–16% of vegetable oil for food and biofuel globally [25, 26]. Different locus mutations leading to chlorophyll deficiency in *B. napus* were identified. Zhu et al. mapped a chlorophyll-deficient mutant to C06, and 18 markers cosegregated with the mutant locus [27]. A dominant chlorophyll-deficient locus was mapped on chromosome C08 spanning a 0.9 cM interval with 22 genes in *B. napus* [28]. Chu et al., using iTRAQ-based quantitative proteomics analysis, identified 443 proteins related to photosynthesis, porphyrin and chlorophyll metabolism, biosynthesis of secondary metabolites, and carbon fixation [29]. The Fv/Fm ratio, qP and electron transport rate in the chlorophyll b-deficient mutant were higher than those in the wild type according to chlorophyll fluorescence and thermoluminescence tests in the chlorophyll b-deficient mutant in *B. napus* [30]. A spontaneous mutant from Qingyou 10 caused by 2 recessive loci and one locus was mapped to A01 in *B. napus* [27]. The chlorophyll-deficient mutant *Cr* was introduced into male sterile lines, and the yellowish color was used as a phenotype marker to produce hybrid seeds [31].

In our research, we identified a chlorophyll-deficient mutant with yellow leaves by using ethyl methanesulfonate (EMS) treatment. The phenotype, photosynthetic characteristics, chloroplast anatomy and expression profile were investigated. Furthermore, the mutant locus was mapped on Chr. A01 by using BSA-Seq.

## Results

### The mutant showed reduced chlorophyll content and weak florescence

**Fig. 1:**
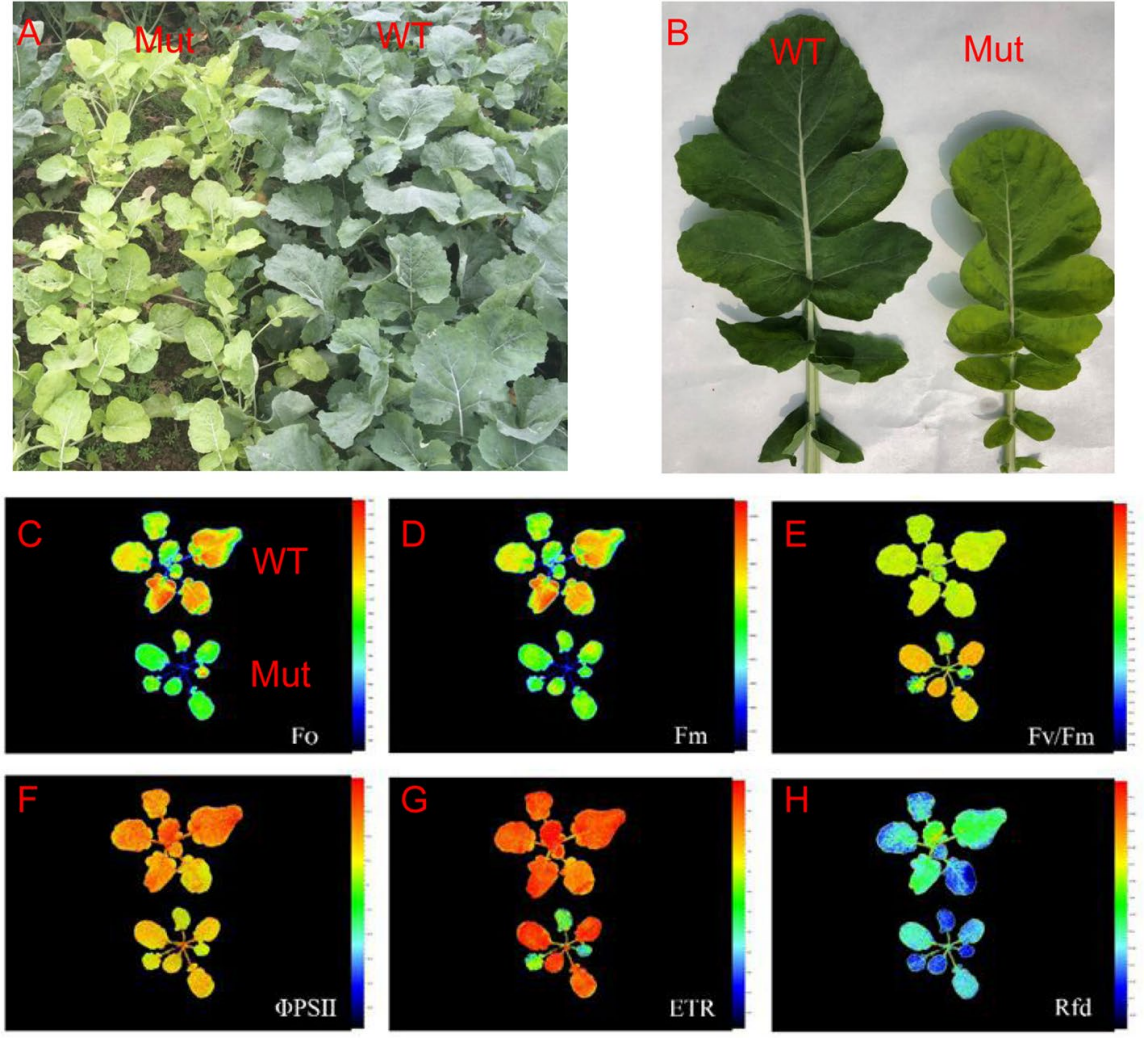
Phenotypic and chlorophyll fluorescence kinetics observations between the mutant type (Mut) and the wild type (WT) at the seedling stage. Note: **A** plants; **B** leaves. **C** to **H** show the fluorescence kinetics of Fo, Fm, Fv/Fm, ΦPSII, ETR, and Rfd, respectively. Top‚ WT; Bottom‚ Mut; Fo, initial fluorescence; Fm, maximum fluorescence; Fv/Fm, PSII maximum light energy conversion efficiency; Φ PSII, actual photochemical efficiency of PSII; ETR, apparent electron transfer efficiency; Rfd, fluorescence decay rate

Compared with the wild type, the mutant leaf displayed a yellow color for the whole growth period (Fig. 1A, B). The mutant plants and seeds were smaller than those of the wild-type plants because of the reduced photosynthetic efficiency. Furthermore, only one-third of the mutant seeds germinated under the usual treatment. The F1 plants displayed a green leaf color, but the segregation did not fit the 3:1 ratio because of the impaired germination of the mutant seeds. The pigment content of the mutant was significantly lower than that of the wild type in fully expanded leaves (Table 1). Chlorophyll fluorescence kinetic tests indicated that the F_0_ and F_m_ in the mutant were weaker than those in the wild type, especially in the fully expanded leaf (Fig. 1C, D). There were no large differences in the remaining florescence characteristics between the mutant and wild type (Fig. 1E-H).

**Table 1:** Photosynthetic pigments content in fully expanded leaves at five-leaf stage between mutant type (Mut.) and wild type (WT)

Lines	Chl.a	Chl.b	Total Chl	Car	Chl.a/Chl.b
Mut	0.45 ± 0.13^**^	0.08 ± 0.02^**^	0.528 ± 0.14^**^	0.119 ± 0.045^*^	5.667 ± 1.086^**^
WT	1.11 ± 0.22	0.28 ± 0.07	1.38 ± 0.25	0.276 ± 0.054	4.125 ± 0.875

^*^Indicates significant difference at *P* < 0.05 level. ** indicates a significant difference at the level of *P* < 0.01

### The mutant had impaired chloroplast development

**Fig. 2:**
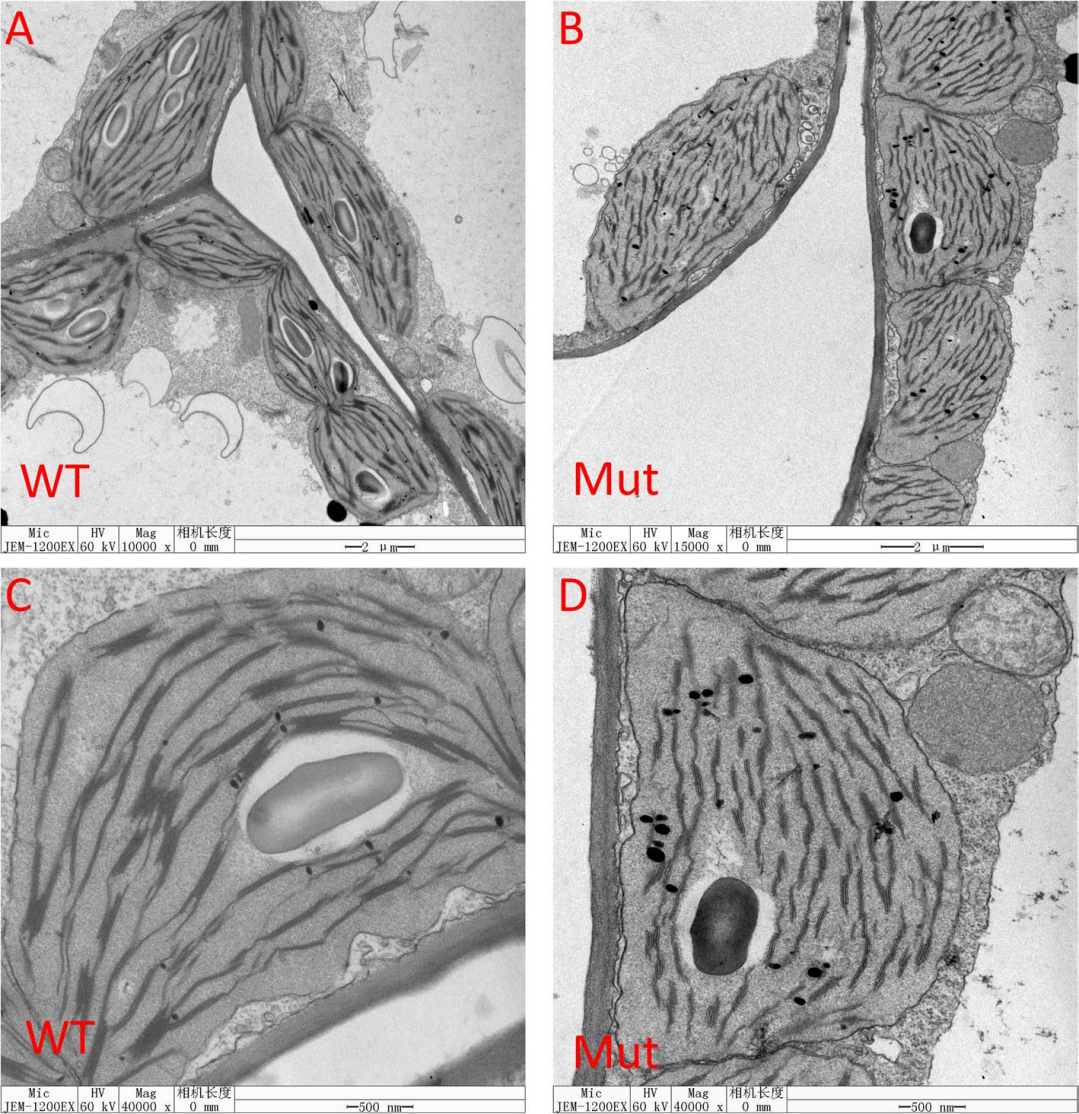
Comparison of the chloroplast ultrastructure of the WT and the Mut. Note: **A** and **C** are wild type; **B** and **D** are mutant; G‚ chloroplast grana lamella; S‚ starch grain; OG‚ osmiophilic granules

There were significant morphological and structural differences between the WT and the mutant. The chloroplasts in the WT were smooth and spindly in shape, with abundant and well-ordered thylakoids. The thylakoids were obviously connected by stromal lamella (Fig. 2 A, C). Compared with the WT, the chloroplast shape was less irregular and less connected between thylakoids (Fig. 2 B, D). Furthermore, there were more osmiophilic granules and smaller starch grains in the mutant than in the WT.

### Chlorophyll and chlorophyll precursors were reduced in the mutant

**Fig. 3:**
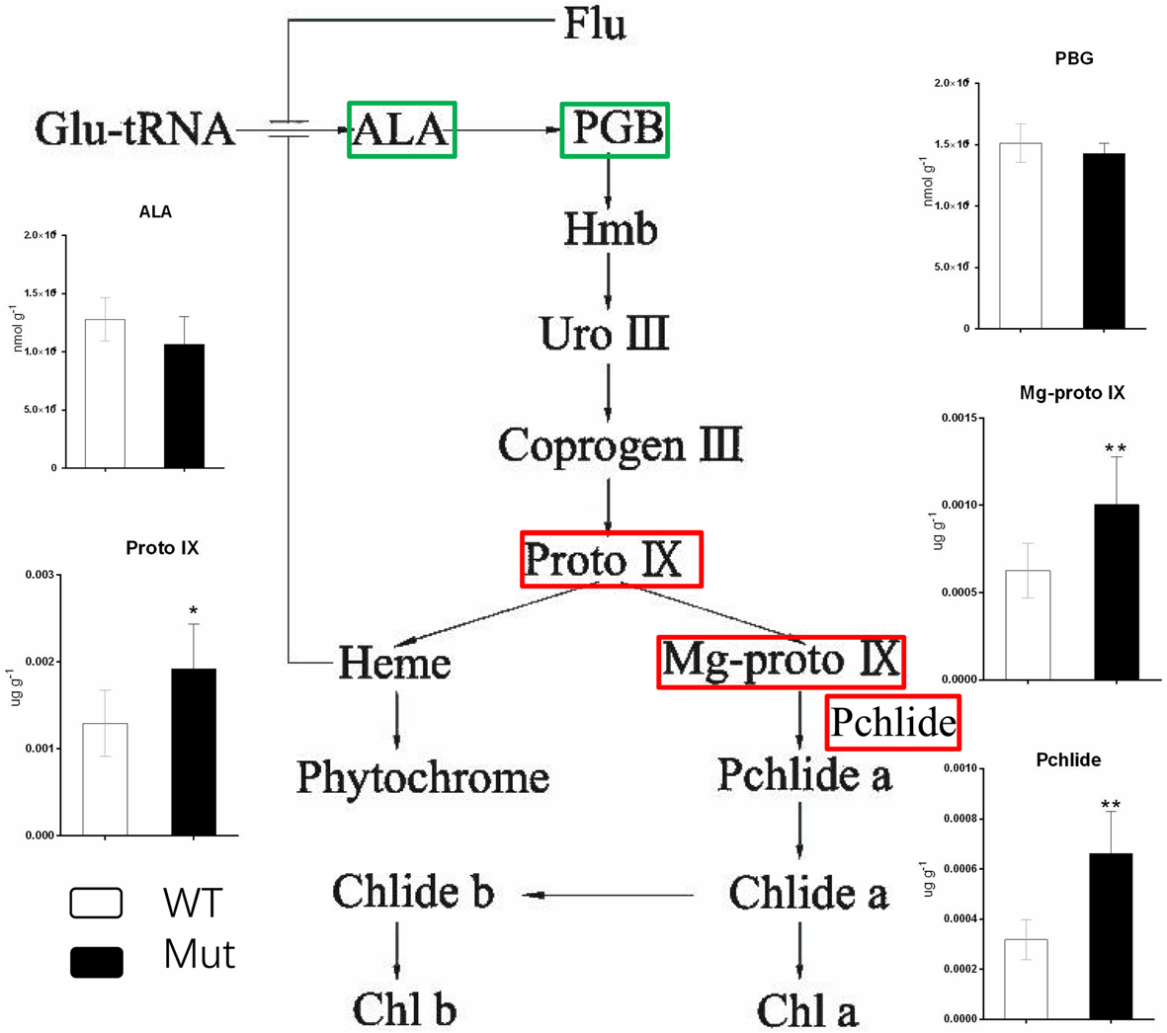
Comparison of chlorophyll synthesis precursor content in wild-type and mutant plants. Note: ALA, δ-aminolevulinic acid; PBG, bilirubin; Proto IX, protoporphyrin IX; Mg-proto IX, magnesium protoporphyrin IX; Pchlide, prophytophyte chlorophyll

The spectrophotometer method was used to determine the contents of chlorophyll and chlorophyll synthetic precursors in mutant and WT leaves. The mutant plant had significantly lower chlorophyll a, chlorophyll b, total chlorophyll, and carotenoid contents than the wild type (Table 1). The contents of δ-aminolevulinic acid (ALA) and porphobilinogen (PBG) of chlorophyll synthetic precursors in the mutant were not significantly different from those in the WT. The contents of tetrapyrroles, such as protoporphyrin IX (Proto IX), magnesium protoporphyrin IX (Mg-proto IX), and prophytophyte chlorophyll (Pchlide), were significantly higher in the mutant than in the WT (Fig. 3). ALA and Pchlide were the early and later precursors in chlorophyll synthesis, respectively, suggesting that chlorophyll synthesis was impaired at the later step of Pchlide conversion into chlorophyll a.

### The mutant had reduced photosynthetic ability

**Fig. 4:**
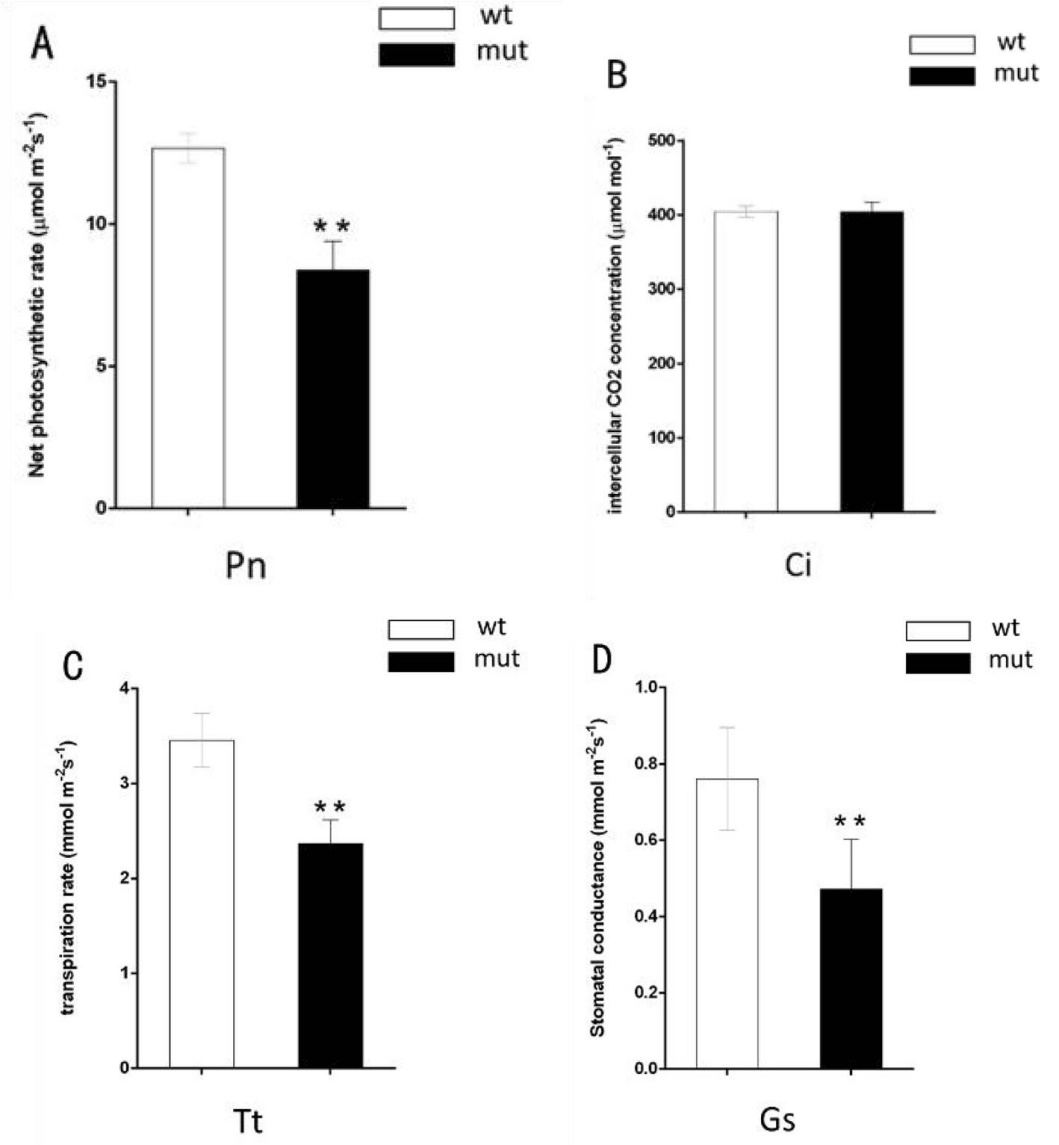
Photosynthetic indices of the WT and the Mut. Note: Pn‚ photosynthetic rate; Ci‚ intercellular CO_2_ concentration; Tt‚ stomatal conductance; Gs‚ transpiration rate. Values are means ± standard error (*n* = 5), **: significantly different at the *P* < 0.01 level

The net photosynthetic efficiency, stomatal conductance and transpiration rate were significantly reduced in the mutant, but the intercellular CO_2_ concentration was similar. The results indicate that the decrease in net photosynthetic efficiency was not due to a stomatal flaw, such as a decrease in stomatal conductance or an insufficient supply of CO_2_, but was instead due to the poor development of chloroplasts inside the mesophyll cells (Fig. 4).

### The mutant locus was mapped on the A01 chromosome

**Fig. 5:**
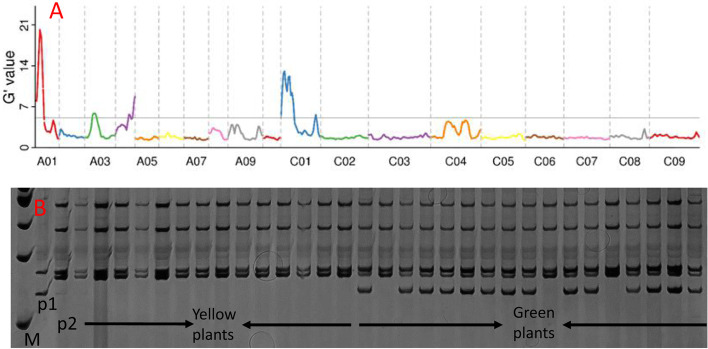
Distribution of G’values on each chromosome (**A**) (*p* = 10 ^−12^) and SSR *bna108* marker pattern in the yellow and green plants (**B**)

BSA-Seq mapped the candidate gene at 2.72 Mb intervals between 3.36 and 6.07 Mb of chromosome A01 (Fig. 5A). Annotation in the candidate region was performed according to the *B. napus* cultivar Darmor-bzh reference genome (https://www.genoscope.cns.fr/brassicanapus/). A total of 502 genes were located in the candidate region, of which 277 genes contained mutated SNP sites that could cause changes in the protein sequence. SSR markers from 2.72 Mb were designed, and the mutant locus was mapped close to marker *bna108* (Fig. 5 B)*.*

### Transcriptome analysis revealed DEGs related to the photosynthetic system

**Fig. 6:**
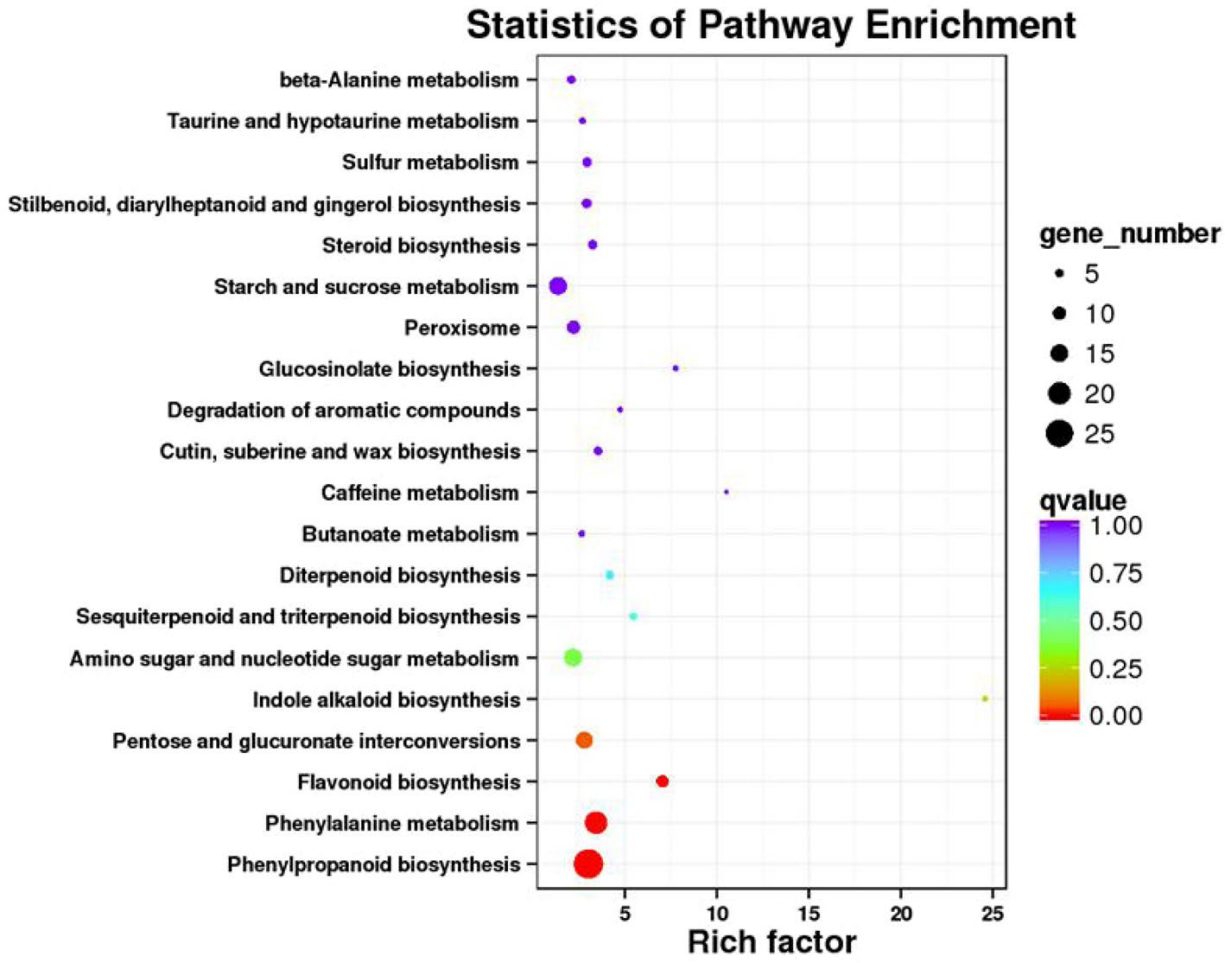
Scatter plot of KEGG pathway enrichment for DEGs

**Fig. 7:**
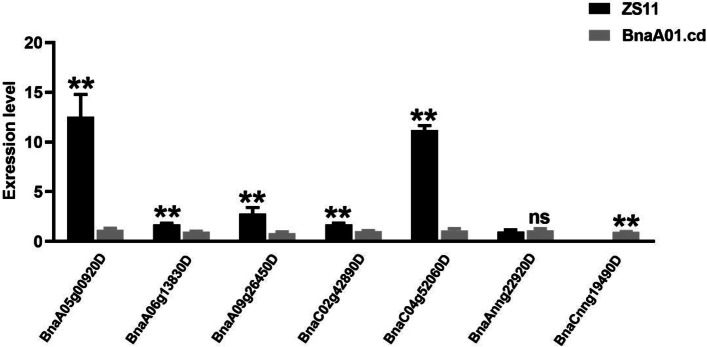
Relative expression levels of DEGs related to the photosynthesis system tested by qRT–PCR in the WT and the Mut

A total of 1273 differentially expressed genes were identified, of which 624 were upregulated and 649 were downregulated. Through KEGG analysis, it was found that the differentially expressed genes were mainly enriched in phenylpropane metabolism and sugar metabolism (Fig. 6). Among the differentially expressed genes related to the photosynthetic system, the expression of the *BnaAnng22920D* gene was severely reduced, and the expression levels of the *BnaC02g42890D* and *BnaCnng19490D* genes were both reduced to zero (Table 2). These genes encode the apoprotein Lhcb that binds to photosynthetic system I (PSI I), important constituent subunits of photosystem I reaction center subunit N (PSAN) and photosystem I reaction center subunit F (PSAF). The qRT–PCR results of the DEGs are shown in Fig. 7. The up- and downregulated genes are listed in supplementary files 2 and 3, respectively.

**Table 2:** Genes related to photosynthesis system in DEGs

DEGs in RNA-Seq	TAIR ID	Gene annotation	FPKM	
			WT	MUT
*BnaAnng22920D*	*AT1G29930*	chlorophyll A/B binding protein 1 (CAB1)	2098.33	469.501
*BnaC02g42890D*	*AT5G64040*	PSAN	958.068	0
*BnaCnng19490D*	*AT1G31330*	photosystem I subunit F (PSAF)	12.5111	0
*BnaA06g13830D*	*AT1G19670*	chlorophyllase 1 (CLH1)	21.4366	3.33526
*BnaA09g26450D*	*AT1G30100*	nine-cis-epoxycarotenoid dioxygenase 5 (NCED5)	1.35018	0.139216
*BnaC04g52060D*	*AT2G46970*	phytochrome interacting factor 3-like 1 (PIL1)	16.09779	0.840105
*BnaA05g00920D*	*AT2G46970*	phytochrome interacting factor 3-like 1 (PIL1)	42.0175	2.2501

## Discussion

In our study, we mapped the mutant locus in Chr. A01, and the plant leaf remained yellow throughout the growth stage. *BnaC.ygl* had yellow green leaves, but the color changed to green at later stages, and the mutant locus was mapped to Chr. C06 [27]. A single dominant leaf color mutant was mapped to Chr. C08 [28]. A yellow-virescent gene regulating chlorophyll biosynthesis was mapped to A03, which was controlled by a single recessive nuclear gene [39]. *BnChd1-1*, one of the two recessive loci involved in chlorophyll biosynthesis in *B. napus,* was mapped to A01 [40]. Compared with the former mapped leaf color mutant in *B. napus*, our mutant was mapped to A01, the same chromosome of *BnChd1-1*, but the *BnaA01.cd* leaf color was more obvious than that of the WT, and there was no curl up in the adult leaf. *Brassica napus* is an allotetraploid, and it is difficult to derive a phenotype mutant because there are more gene family members than in diploid crops. Currently, there are fewer leaf color mutants in *B. napus* than in rice; thus, our mutant line and work will enrich leaf mutant resources, which will facilitate chloroplast development research.

Chlorosis is a phenotype of yellowish or light green leaf color and can be a genetic mutation or a sign of nutritional deficiency. The genetic mutation causing chlorosis in *B. napus* might only occur in the seedling stage or during the whole growth stage. All chlorosis showed reduced Chl. content and impaired light energy absorption during photosynthesis. Chlorophyll synthesis starts from the precursor glutamyl-tRNA, branched at protoIX. Magnesium chelatase, the key enzyme in this pathway, inserts magnesium atoms into protoIX synthesis, the direct precursor of Chl. a and Chl. b. In our chlorophyll synthetic precursor test, the contents of δ-aminolevulinic acid (ALA) and porphobilinogen (PBG) showed no significant difference between the mutant and WT, but the contents of Proto IX, Mg-proto IX, and Pchlide were significantly higher in the mutant. The accumulation of Proto IX, Mg-proto IX, and Pchlide not only impairs chlorophyll synthesis but might also increase ROS production under light illumination, which would be toxic for chlorophyll development [41, 42]. In the mutant, the chloroplast structure was dramatically changed, including an oval shape, fewer stromal lamellae and thylakoids, and more osmiophilic granules. These changes occurred in senescent leaves. In the moth orchid leaf mutant, the green leaf sector with typical chloroplasts, including well-organized thylakoids and starch grains, but the yellow leaf area with poor developed plastids and more osimiophilic granules [43]. There are more grana in the yellow-white leaf at advanced senescence compared with leaf for 6 days regreening [44]. In rice early senescence 2 (es2) mutant, more osmiophilic granules (OG) were found in es2 compared to wild type [45]. The SEM observations indicate that the mutant chloroplasts from young leaves might be in the senescence state.

BSA-Seq mapped *BnaA01.cd* in A01, spanning a 2.72 Mb region, which included approximately 277 genes with SNPs. For candidate gene identification, more studies are needed for large population construction. The transcriptome data provide information about the leaf expression profile, which explains the phenotype and functional changes in the mutant. The DEGs were enriched in phenylpropane metabolism and the sugar synthetic system. The gene *BnaAnng22920D,* homologous to *AT1G29930*, encoding chlorophyll A/B-binding protein 1 (CAB1), was significantly downregulated. CAB1 binds chlorophyll from the LCHP complex [46], and CAB1 reduction leads to free chlorophyll degradation [47]. Furthermore, a reduction in the LCHP complex will indirectly influence thylakoid membrane structure and state transition. Two strongly downregulated genes, *BnaC02g42890D* and *BnaCnng19490D,* homologous to *AT5G64040* and *AT1G31330*, encode the proteins PSAN and PSAF, respectively. PSAN functions in mediating the binding of the antenna complexes to the PSI reaction center and the core antenna, docking plastocyanin to the PSI complex [48]. PSAF participates in the efficiency of electron transfer from plastocyanin to P700 [49]. The above protein reduction will impair electron transfer and energy transition.

## Conclusions

A mutant exhibiting chlorophyll deficiency was identified in *B. napus*, and the mutant locus was mapped on A01 in the 2.72 Mb region. The mutant chlorophyll content and photosynthetic efficiency were significantly reduced compared with those of the wild type. Abnormal chloroplasts and thylakoids less connected to the stroma lamella appeared in the mutant. This work on the mutant will provide more genetic and physiological information concerning chloroplast development in *B. napus*.

## Methods

### Plant materials

A chlorophyll-deficient mutant (*B. napus* chlorophyll deficient, *BnaA01.cd*) was screened from the EMS-mutated population. The mutant population was constructed from the inbreed line Zhongshuang11 (ZS11), and seeds were soaked in 1% EMS solution for 12 h. A mutant with yellow leaves was identified in the M2 generation and selfed for phenotype validation and crossing in the next generation. The inbred lines ZS11 and *BnaA01.cd* were used for phenotype comparison. Field growth was the same as usual.

### Kinetic chlorophyll fluorescence imaging

Plants grown in climate chambers until the 7-leaf stage were used for fluorescence imaging. Chlorophyll fluorescence imaging was performed using a FluorCam PlantScreen™ phenotyping system (Photon Systems Instruments, Czech Republic). Actinic light at 150 μmol of photons·m^−2^·s^−1^ and saturating pulse intensity at 100% were applied to 16-h dark-adapted plants. The chlorophyll fluorescence levels of the wild-type and mutant plants were imaged based on the parameters F_0_, Fm, Fv/Fm, PSII, ETR and Rfd.

### Transmission electron microscopy

Leaf samples of wild-type ZS11 and mutant *BnaA01.cd* from the bolting stage were prepared by transmission electron microscopy. Leaves were cut into 0.5 × 0.5 cm sections and fixed in 2.5% (w/v) glutaraldehyde in 0.1 M phosphate buffer (pH 7.4) at 4 ºC for 4 h. Samples were further fixed in 1% OsO4 using the same buffer for 12 h. Ultrathin sections of the samples and transmission electron microscopy observations were performed according to Yi et al. [32].

### Chlorophyll and precursor content determination

Fresh leaves of ZS11 and *BnaA01.cd* mutant plants were sampled from the bolting stage and used to measure chlorophyll content according to the method described by Wu et al. [33]. The same leaf was used for the 5 precursor content determinations. The precursor δ-aminolevulinic acid (ALA) content was extracted and measured following Dei [34]. The determination of bilirubin (PBG) was performed according to [35]. The Proto IX, Mg-Proto IX, and Pchlide precursors were extracted and measured according to the methods described by Rebeiz et al. [36].

### Photosynthetic performance test

The leaves from the bolting stage were used for net photosynthetic efficiency, stomatal conductance, transpiration rate and intercellular CO_2_ concentration tests using an LI-6400 (LI-6400, Li-Cor, Lincoln, NE) according to the manual. Measurements were conducted at a saturating light intensity of 1,500 μmol photons m^−2^ s^−1^, with an air flow of 500 μmol air s^−1^ and a fixed flux of 400 μmol CO_2_ mol^−1^ air. Each genotype with 10 plants and 3 fully expanded leaves was measured for each plant on a sunny day at noon, and the mean value was used for data analysis.

### BSA-Seq and SSR mapping

A cross was made between Z300 (line with normal green leaf) and the *BnaA01.cd* to create F_1_ plants which were subsequently selfed to create a F_2_ population with 322 plants. The yellow and green leaf bulks were prepared from the segregated F_2_ population, each containing 15 plants with distinctive leaf color. The two parents and two DNA bulks were sent to GENOSEQ (Wuhan, China) for BSA-Seq. Sequence library construction and SNP calling were performed as described by Zhao et al. [37].

Based on the BSA-Seq interval, 64 SSR primer pairs were developed to confirm the BSA-Seq mapped region. The SSR loci were searched by the Tandem repeats finder [38] with a maximum of 6-bp motifs and a minimum of 3 repeats, and the polymorphic SSR primers are provided in supplementary file 1. The test was performed by standard PCR with primer pair-specific annealing temperature followed by PAGE gel electrophoresis.

### RNA extraction and transcriptome analysis

The yellow and green leaves from mutant and wild-type plants at the bolting stage were sampled for RNA extraction and cDNA library construction, with two biological repeats. The leaves were kept in liquid nitrogen for immediate RNA extraction. Total RNA was extracted using TRIzol Reagent and treated with RNase-free DNase I. cDNA library construction and sequencing on the Illumina HiSeq 4000 platform were performed by Biomarker Technologies (Beijing, China). After the removal of low-quality reads, the clean reads were aligned to the *B. napus* reference genome. Fragments per kilobase million (FPKM) values were calculated to estimate gene expression levels. FDR < 0.001 and fold change ≥ 4 were used to assess the significance of differences in gene expression. qRT–PCR primers were designed using Primer Premier 5 and synthesized by Sangon Biotech (Shanghai) Co. qRT–PCR was performed using a Bio–Rad CFX96 Real-time System with SYBR® Green PCR Supermix (CA, USA) in triplicate. BnActin7 was used as a control. Each reaction included 10 µl SYBR ® Green PCR Supermix, 0.4 µL primer F, 0.4 µL primer R, 7.2 µL ddH_2_O, and 2 µL cDNA template.

## Supplementary Information

**Additional file 1. Table S1.** Polymorphic SSR primers in the BSA-Seq region.

**Additional file 2.**

**Additional file 3.**

## Data Availability

All data are presented in the main manuscript and the additional supporting files. The Kanehisa laboratory have provided us permission of The KEGG pathway database from www.kegg.jp/feedback/copyright.html [50]. KOBAS software was used to test the statistical enrichment of differential expression genes in KEGG pathways [51]. ZS11 was kindly provided by Dr. Jiaqin Shi, oil crop research institute, CAAS, Wuhan, China.
