# Supplementary material for: Genetic mapping and physiological analysis of chlorophyll-deficient mutant in Brassica napus L
Source: BMC Plant Biol. 2022 May 18;22:244. doi: 10.1186/s12870-022-03630-9 (PMC9115954; doi:10.1186/s12870-022-03630-9)
Supplement: Supplementary file 1 — Additional file 1. Table S1. Polymorphic SSR primers in the BSA-Seq region. [file 12870_2022_3630_MOESM1_ESM.docx]

Table 1 Polymorphic SSR primers in the BSA-Seq region

| Primer | Forward Primer sequence(5’→3’) | Reverse Primer sequence(5’→3’) |
| --- | --- | --- |
| *bna108* | CAAACACGCGATCTCTCTAACA | GAACAACAATTCGAGGTGCATA |
| *bna206* | TTGTTTAGGGGCTGTTTAGACG | GCCAATCTCTTTAGGCGAATTA |
| *bna502* | TCTTGTTCTTCCCTCTCTTTGC | CCCCACAGGTACACACACATAC |
| *bna503* | CACTAATTTGAACAGAGCCGGT | TAAAATACATCCCGTCCGTAGG |
